# Natural Products from *Physalis alkekengi* L. var. *franchetii* (Mast.) Makino: A Review on Their Structural Analysis, Quality Control, Pharmacology, and Pharmacokinetics

**DOI:** 10.3390/molecules27030695

**Published:** 2022-01-21

**Authors:** Jing Yang, Yanping Sun, Feng Cao, Bingyou Yang, Haixue Kuang

**Affiliations:** 1 Key Laboratory of Basic and Application Research of Beiyao, Ministry of Education, Heilongjiang University of Chinese Medicine, Harbin 150040, China; mayday111@163.com (J.Y.); 18704608056@163.com (Y.S.); ybywater@163.com (B.Y.); 2 Ganjiang Chinese Medicine Innovation Center, Nanchang 330000, China; 18435166854@163.com

**Keywords:** the calyxes and fruits of *P. alkekengi*, structural analysis, quality control, pharmacology, pharmacokinetics

## Abstract

The calyxes and fruits of *Physalis alkekengi* L. var. *franchetii* (Mast.) Makino (*P. alkekengi*), a medicinal and edible plant, are frequently used as heat-clearing and detoxifying agents in thousands of Chinese medicine prescriptions. For thousands of years in China, they have been widely used in clinical practice to treat throat disease, hepatitis, and bacillary dysentery. This systematic review summarizes their structural analysis, quality control, pharmacology, and pharmacokinetics. Furthermore, the possible development trends and perspectives for future research studies on this medicinal plant are discussed. Relevant information on the calyxes and fruits of *P. alkekengi* was collected from electronic databases, Chinese herbal classics, and *Chinese Pharmacopoeia*. Moreover, information was collected from ancient documents in China. The components isolated and identified in *P. alkekengi* include steroids, flavonoids, phenylpropanoids, alkaloids, nucleosides, terpenoids, megastigmane, aliphatic derivatives, organic acids, coumarins, and sucrose esters. Steroids, particularly physalins and flavonoids, are the major characteristic and bioactive ingredients in *P. alkekengi*. According to the literature, physalins are synthesized by the mevalonate and 2-C-methyl-d-erythritol-4-phosphate pathways, and flavonoids are synthesized by the phenylpropanoid pathway. Since the chemical components and pharmacological effects of *P. alkekengi* are complex and varied, there are different standards for the evaluation of its quality and efficacy. In most cases, the analysis was performed using high-performance liquid chromatography coupled with ultraviolet detection. A pharmacological study showed that the crude extracts and isolated compounds from *P. alkekengi* had extensive in vitro and in vivo biological activities (e.g., anti-inflammatory, anti-tumor, immunosuppressive, antibacterial, anti-leishmanial, anti-asthmatic, anti-diabetic, anti-oxidative, anti-malarial, anti-Alzheimer’s disease, and vasodilatory). Moreover, the relevant anti-inflammatory and anti-tumor mechanisms were elucidated. The reported activities indicate the great pharmacological potential of *P. alkekengi*. Similarly, studies on the pharmacokinetics of specific compounds will also contribute to the progress of clinical research in this setting.

## 1. Introduction

**Figure 1:**
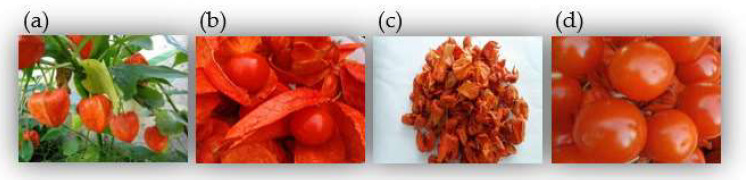
Images of *P. alkekengi*. (**a**) The whole plant; (**b**) Calyxes and fruits; (**c**) Calyxes; (**d**) Fruits.

*P. alkekengi* is a perennial plant (Figure 1a) belonging to the genus *Physalis* of the family Solanaceae. The calyxes and fruits of *P. alkekengi* (known as Jindenglong in Chinese) (Figure 1b) are distributed in Europe and Asia. The use of the calyxes and fruits of this plant was first recorded in the prestigious monograph *Shennong Bencao Jing* in China [1]. Subsequently, it was included as an important traditional Chinese medicine (TCM) in the *Ben Cao Gang Mu* and pharmacopoeia [2]. Calyxes are green, self-expanded into an oocyst shape, slightly concave at the base, 2.5–5 cm in length, 2.5–3.5 cm in diameter, have thin leathery skin, and are orange-red or fire-red when mature (Figure 1c). Fruits are spherical, orange-red, and 10–15 mm in diameter (Figure 1d). This plant has been used for >2000 years in China, and its activities have been defined as “heat-clearing and detoxifying, relieving sore throat to reducing phlegm and inducing diuresis for treating strangurtia” in TCM theory [3,4]. In clinical practice, *P. alkekengi* is often used in combination with other TCMs for the treatment of cough, excessive phlegm, pharyngitis, sore throat, dysuria, pemphigus, eczema, and jaundice [5]. Currently, the 12 TCM formulae and modern pharmaceutical preparations of the calyxes and fruits of *P. alkekengi* are listed in the Pharmacopoeia of the People’s Republic of China and used in folk medicine [6]. For example, qing guo ointment, a TCM formula composed of seven medicinal herbal plants (i.e., the calyxes and fruits of *P. alkekengi*, *Cannarii Fructus*, *Sophorae Tonkinensis* Radix et Rhizoma, *Sterculiae Lychnophorae Semen*, *Trichosanthis Radix*, *Ophiopogonis Radix*, and *Chebulae Fructus*), is effective for clearing the throat and quenching thirst, treating aphasia and hoarseness, and relieving sore throat, dry mouth, and dry tongue [1].

In the last decades, reviews concerning research progress on the calyxes and fruits of *P. alkekengi* have been published, mainly focusing on the chemical components, traditional uses, toxicology, and pharmacological activities [6]; however, thus far, there are no reports on structural analysis, quality control, and pharmacokinetics. In recent years, new pharmacological activities have been discovered, and the main active ingredients in *P. alkekengi* are physalins and flavonoids [7]. Therefore, we herein provide a literature review on the structural analysis of physalins and flavonoids in the calyxes and fruits of *P. alkekengi*. We have also prepared a comprehensive and up-to-date report for the known pharmacological activities. In addition, the quality control and pharmacokinetics studies are summarized in detail. We hope that the current review will provide a theoretical basis and valuable data for future in-depth studies and the development of useful applications.

## 2. Structural Analysis

### 2.1. Physalins

*P. alkekengi*, a high-value multipurpose medicinal plant, is a rich reservoir of structurally diverse and biologically active terpenoids, termed physalins. Thus far, >70 physalin-type natural products have been isolated; most of them possess a 13,14-seco-16,24-cycloergostane skeleton, with anolides with a C-22, C-26 δ-lactone side chain or C-23, C-26 γ-lactone side chain of C28 ergostane-type steroids [8,9]. According to the bonding type of C-14, physalins can be divided into two subtypes: physalins (Type I), in which C-14 is connected to C-17 through oxygen to form an acetal bridge, and neophysalins (Type II), where C-14 is connected to C-16 and C-15/C-17 is esterified to form lactone [10].

**Figure 2:**
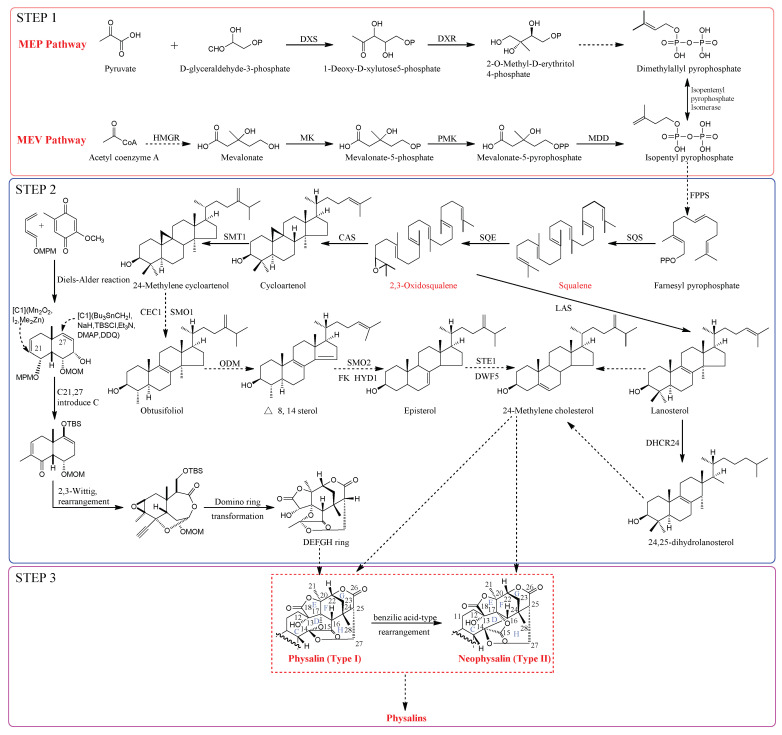
An overview of physalin synthesis in the calyxes and fruits of *P. alkekengi*. Solid one-headed arrows indicate single-step irreversible reactions, while dotted arrows indicate several steps of reactions. Abbreviations: Bu, butyl; CAS, cycloartenol synthase; CEC1, cycloeucalenol cycloisomerase; CoA, coenzyme A; DDQ, 2,3-dichloro-5,6-dicyano-1,4-benzo-quinone; DHCR24, 24-dehydrocholesterol reductase; DMAP, 4-dimethylaminopyridine; DWF5, sterol delta-7 reductase; DXR, 1-deoxy-d-xylulose-5-phosphate reductase; DXS, 1-deoxy-d-xylulose-5-phosphate synthase; Et, C2H5; FK, delta 14-sterol reductase; FPPS, farnesyl diphosphate synthase; HMGR, 3-hydroxy-3-methylglutaryl-coenzyme A reductase; HYD1, C-7,8 sterol isomerase; LAS, lanosterol synthase; MDD, mevalonate diphosphosphate decarboxylase; Me, CH3; MEP, 2-C-methyl-d-erythritol-4-phosphate; MEV, mevalonate; MK, mevalonate kinase; MOM, methoxymethyl; MPM, para-methoxyphenylmethyl; ODM, obtusifoliol-14-demethylase; PMK, phosphomevalonate kinase; SMO1, sterol-4α-methyl oxidase 1; SMO2, sterol-4α-methyl oxidase 2; SMT1, sterol methyl transferase 1; SQS, squalene synthase; SQE, squalene epoxidase; STE1, C-5 sterol desaturase; TBS, tert-butyldimethylsilyl.

As shown in Figure 2, the synthesis of physalins can be divided into three steps. In step 1,5-carbon precursor isopentenyl diphosphate and dimethylallyl pyrophosphate are synthesized via cytosolic mevalonate (MEV) and plastid localized 2-C-methyl-d-erythritol-4-phosphate (MEP) pathways, respectively; this is the first step toward the synthesis of physalins in plants [11]. In addition, 1-deoxy-d-xylulose-5-phosphate reductase and 3-hydroxy-3-methylglutaryl-coenzyme A reductase are key enzymes that regulate the MEP and MEV pathways, respectively [12]. In step 2, farnesyl pyrophosphate synthase catalyzes the conversion of isopentenyl diphosphate and dimethylallyl diphosphate to farnesyl pyrophosphate [13]. Additionally, farnesyl pyrophosphate is converted to 24-methylene cholesterol under the action of enzymes (squalene synthase, squalene epoxidase, cycloartenol synthase, etc.). It was also confirmed that cycloartenol, cycloeucalenol, and obtusifoliol are primary intermediates in the synthesis of the 24-methylene cholesterol [14,15]. In step 3, the skeletons of physalins (Types I and II) were produced from 24-methylene cholesterol [13,16,17]. Furthermore, the racemic DEFGH-ring moiety of physalins (Type I) is synthesized through enzymatic kinetic resolution [18]. The intermediate physalins were synthesized by domino ring transformation, a reoptimization of the 2,3-wittig rearrangement, and methylation steps. The DEFGH ring moiety was synthesized from precursors 2-methoxy-5-methylcyclohexa-2,5-diene-1,4-dione and (E)-1-((buta-1,3-dien-1-yloxy)methyl)-4-methoxybenzene and an intermediate of a-allylic alcohol [19,20]. Additionally, physalins can be converted to neophysalins through an acid-induced benzilic acid-type rearrangement reaction [10].

**Figure 3:**
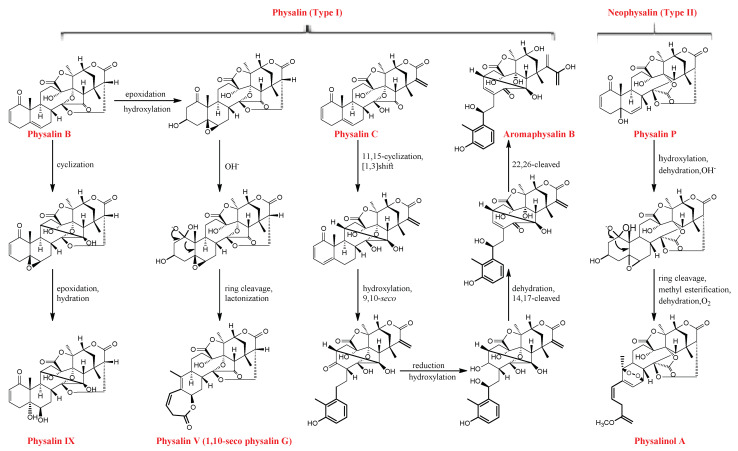
Biogenetic pathway of physalins in the calyxes and fruits of *P. alkekengi*.

The skeletons of physalins, through various biochemical reactions (i.e., desaturation, methylation, hydroxylation, epoxidation, cyclization, chain elongation, and glycosylation), lead to the production of various physalins [21]. As shown in Figure 3, Wu et al. [10] proposed the plausible biogenetic pathway for physalin IX, physalin V, aromaphysalin B, and physalinol A. For example, the epoxidation and hydroxylation of physalin B could produce an intermediate, which could be further ring-cleaved between C-1 and C-10, subjected to lactonization, and dehydrated to yield physalin V. Meanwhile, the cyclization of physalin B between C-11 and C-15 afforded the intermediate; subsequently, the intermediate was further epoxidized and hydrated to obtain physalin IX.

### 2.2. Flavonoids

**Figure 4:**
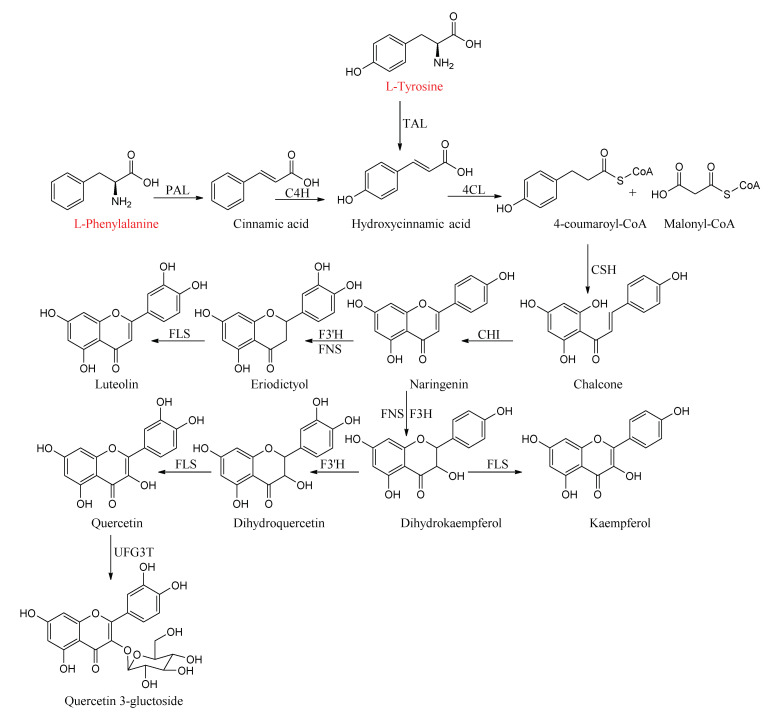
An overview of flavonoid synthesis in the calyxes and fruits of *P. alkekengi.* Abbreviations: 4CL, 4-coumaryl-CoA ligase; C4H, cinnamate-4-hydroxylase; CHI, chalcone isomerase; CHS, chalcone synthase; CoA, coenzyme A; PAL, phenylalnine ammonialyase; F3’H, flavanone 3’-hydroxylase; FLS, flavonol synthase; FNS, flavone synthase; TAL, tyrosine ammonialyase; UFG3T, uridine diphosphate-glucose: flavonoid-3-*O*-glucosyltransferase.

Flavonoids are the second major component of *P. alkekengi*, with a common C6–C3–C6 tricyclic skeleton [22,23]. The main flavonoid synthetic pathway has been characterized in *P. alkekengi* (Figure 4). The C6–C3–C6 carbon backbone was first synthesized through the phenylpropanoid pathway, transforming phenylalanine into 4-coumaroyl-coenzyme A, which finally enters the flavonoid synthesis pathway [24,25]. Next, 4-coumaroyl-coenzyme A combines with three molecules of malonyl-coenzyme A to yield naringenin, which is the source of all flavonoids. Chalcone synthase and chalcone isomerase are the enzymes involved in the two-step condensation [26,27,28]. Naringenin is subsequently converted to luteolin through two reactions catalyzed by flavanone 3’-hydroxylase and flavonol synthase. At the same time, the conversion of naringenin by flavanone 3-hydroxylase yields dihydrokaempferol that can be hydroxylated on the 3’ position of the B-ring by flavanone 3’-hydroxylase, thereby producing dihydroquercetin. The subsequent steps of dihydrokaempferol and dihydroquercetin produce kaempferol and quercetin by flavonol synthase, respectively. The last step of quercetin for the formation of stable compounds involves glycosylation by the enzyme uridine diphosphate-glucose: flavonoid-3-*O*-glucosyltransferase. Finally, quercetin is further converted to quercetin-3-glucoside [29,30].

## 3. Quality Control

The quality control of calyxes and fruits is extremely important for their use. Extensive studies evaluated methods for the analysis of calyxes and fruits. According to the *Chinese Pharmacopoeia*, and based on the morphological, microscopic, and high-performance liquid chromatography (HPLC) analysis and thin-layer chromatography (TLC) identification, the minimum content of luteoloside for qualifying the calyxes and fruits of *P. alkekengi* is ≥0.10% [1]. However, due to the complex chemical components and diverse pharmacological activities of herbal medicines, a single quantitative marker appears to be insufficient for the assessment of quality. Currently, multiple compounds (mainly physalins, flavonoids, and polysaccharides) have been used to validate the quality of this herb by TLC, HPLC, and ultra-performance liquid chromatography-mass spectrometry (UPLC/MS) [31,32].

To obtain reliable pharmacological effects, the concentration of the chemical components of calyxes and fruits should be controlled; it is mainly determined by the season and harvesting time, as well as climatic and geographical conditions [33,34]. The effective composition of calyxes significantly changes with the growth period during harvest. A study showed that the content of physalin D in immature calyxes (0.7880 ± 0.0612%) was four-fold higher than that measured in mature calyxes (0.2028 ± 0.016%). Of note, the content of physalin D in fruits was markedly lower (immature fruits: 0.0992 ± 0.0083%; mature fruits: 0.0259 ± 0.0021%) [35]. Kranjc et al. [36] developed the first high-performance thin-layer chromatography (HPTLC) and HPTLC-MS/MS methods that can characterize physalins in crude extracts obtained from different parts of *P. alkekengi* at different stages of maturity. These findings indicated that only certain parts of the plant are appropriate for specific pharmaceutical applications. The HPTLC method overcame some of the drawbacks in analytical physalin profiling, providing a new approach to quality control for *P. alkekengi*. In addition, 31 samples of *P. alkekengi* collected from different habitats were analyzed using fingerprinting. The results showed that the contents of Baishan, Xinxiang, and Shenyang differed considerably [37]. Huang et al. [38] reported the fragmentation behavior of major physalins in *P. alkekengi* calyxes via ultra-high performance liquid chromatography (UHPLC)-quadrupole time of flight tandem mass spectrometry (QTOF-MS/MS). The content of 4,7-didehydroneophysalin B in fruits and calyxes of *P. alkekengi* was determined by HPLC and UPLC-MS/MS. The results showed that the contents of 4,7-didehydroneophysalin B were 2.18% (50% ethanol extract), 0.42% (70% ethanol extract) by HPLC, and 15.75–70.88 μg/g by UPLC-MS/MS. The results suggested that the content of 4,7-didehydroneophysalin B is relatively high and could be used as an index for the quality control of the medicinal material [39,40,41]. The contents of luteoloside, polysaccharides, reducing sugar, lutein, and *β*-carotene in samples obtained from different habitats were compared, revealing significant differences [42,43,44]. Moreover, some researchers have determined the contents of luteolin and luteoloside in pharmaceutical preparations (i.e., *Physalis permviana* liquid and Jinhuang yanyan tablets) [45,46]. These data provided an important theoretical basis for the harvesting of *P. alkekengi*, identification of physalins, and evaluation of the clinical applications of this medicinal herb. However, wide variations were observed in the contents of these compounds due to differences in the sources and time points of sample collection. Furthermore, the fruits of *P. alkekengi* also contained organic acids and smaller amounts of (hydroxy)cinnamoyl hexosides and amino acids [5]. The quantitative analysis of *P. alkekengi* is shown in Table 1.

## 4. Pharmacology

Pharmacological experiments showed that the various crude extracts and compounds isolated from *P. alkekengi* have diverse biological activities (e.g., anti-inflammatory, anti-tumor, immunosuppressive, anti-microbial, anti-leishmanial, anti-asthmatic, anti-diabetic, etc.). In addition, the mechanisms of action of the anti-inflammatory and anti-tumor activities were also reported. The main pharmacological activities of crude extracts and compounds are shown in Table 2.

### 4.1. Anti-Inflammatory Activity

Studies involving in vitro and in vivo models of lipopolysaccharide-stimulated (LPS-stimulated) THP-1 cells, mouse ear-swelling, rat cotton pellet granuloma, and rat hind paw edema have confirmed that ethanol and methanol extracts from *P. alkekengi* calyxes exert anti-inflammatory effects. The extracts achieve these effects by inhibiting the production of nitric oxide (NO), prostaglandin E2 (PGE2), tumor necrosis factor-α (TNF-α), interleukin-1 (IL-1), inducible nitric oxide synthase (iNOS), and cyclooxygenase-2 (COX-2) [62,91]. As active ingredients isolated from *P. alkekengi*, physalins A, B, D, E, F, H, G, L, O, V, D1, X, VII, and I, isophysalin A, isophysalin B, and aromaphysalin B showed anti-inflammatory activity. At a concentration of 20 µM, physalins A, O, L, and G and isophysalin A inhibited the LPS-induced NO production by blocking TNF-α [47,48]. Physalins B, E, F, G, H, V, X, D1, VII, and I, isophysalin B, and aromaphysalin B reduced the levels of proinflammatory mediators NO, TNF-α, IL-6, IL-12, and interferon-γ (IFN-γ) in LPS-stimulated and IFN-γ-stimulated macrophages, RAW 264.7 cells, and 12-*O*-tetradecanoylphorbol-13-acetate (TPA)- and oxazolo-induced dermatitis. These effects occurred through upregulation of the signal transducer and activator of transcription 6 (STAT6) and downregulation of nuclear factor-κB (NF-κB) and the STAT1 signaling pathway [48,49,51,52,53,55,56]. The anti-inflammatory effects of four flavonoids (i.e., luteolin, apigenin, kaempferol, and quercetin) were related to inhibition of the production of NO, IL-6, IL-12, TNF-α, STAT-1, and NF-κB, the expression of C–C motif chemokine ligand 2/monocyte chemoattractant protein-1 (CCL2/MCP-1) and C–X–C motif chemokine ligand 1/KC (CXCL1/KC), and paw edema [57,58,59,60]. Ombuine inhibited the production of NO in LPS-damaged macrophage cells, with a half maximal inhibitory concentration (IC_50_) value of 2.23 ± 0.19 µM [57].

### 4.2. Anti-Tumor Activity

Recently, in vitro experimental studies showed the anti-tumor activity of physalins in non-small cell lung cancer, human melanoma A375-S2 cells, and tumor cell lines (A549, K562). The results indicated that physalins A and B have strong anti-tumor activity and induced G2/M cell cycle arrest in non-small cell lung cancer and A375-S2 cells. The mechanism involved in this effect is related to the inhibition of Janus kinase 2 (JAK2) phosphorylation, JAK3 phosphorylation, both constitutive and induced STAT3, reactive nitrogen species (RNS), reactive oxygen species (ROS), and cyclin-dependent kinase 1/cyclin B1 (CDK1/CCNB1) complex, as well as the promotion of the p53-NADPH oxidase activator- (p53-NOXA), p38-NF-κB, and p38 mitogen-activated protein kinase/ROS (MAPK/ROS) pathways [66,67,69,70,72,73]. Physalin A also increased the content of detoxifying enzyme in HepG2 cells, induced apoptosis in HT1080 cells, and inhibited growth in prostate cancer cells (CWR22Rv1 and C42B). These effects occurred by activating the nuclear factor erythroid 2-related factor 2–antioxidant response element (Nrf2–ARE), death receptor apoptotic, JUN N-terminal kinase (JNK), and extracellular signal-regulated kinase (ERK) signaling pathway; the IC_50_ values were 20, 10.7, 14.2, and 1.9–4.3 μM, respectively [65,67,68,71]. In addition, six types of cancer cells (i.e., prostate, human HCT116 colon, human DLD-1 colon, breast, TNF-α-stimulated HeLa, and human T cell leukemia Jurkat) were treated with physalin B. The treatment inhibited the activation of TNF-α-induced NF-κB and phorbol 12-myristate 13-acetate (PMA)-induced NF-κB pathways, whereas it promoted the activation of ERK, JNK, p38 MAPK, and P53 pathways [9,74,75,76,79]. Physalin F decreased the TOPFlash reporter activity, inhibited the effects on T-47D cells, and induced cell apoptosis via ROS-mediated mitochondrial pathways [80,81,82].

**Figure 5:**
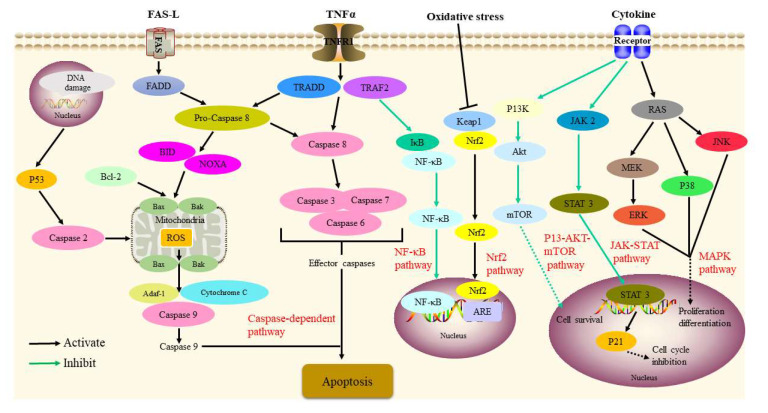
Signaling pathways involved in the antitumor activity of *P. alkekengi* and its constituents.

In vivo, physalins A and F clearly inhibited tumor growth by downregulating β-catenin in xenograft tumor-bearing mice [66,80]. At 10 mg/kg and 25 mg/kg, respectively, physalins B and D inhibited tumor proliferation in mice bearing sarcoma 180 tumor cells [78]. In short, the anti-tumor activity of *P. alkekengi* and its constituents was associated with the downregulation of JAK/STAT3, TNF-α-induced NF-κB, PMA-induced NF-κB, and phosphoinositide-3-kinase-Akt-mechanistic target of the rapamycin (PI3K/Akt/mTOR) signaling pathway. Moreover, it was linked to the upregulation of the death receptor apoptotic, p53-NOXA, p38-NF-κB, p38 MAPK/ROS, p21, and Nrf2 signaling pathway. The signaling pathways are given in Figure 5.

### 4.3. Immunosuppressive Activity

The immunosuppressive activity of *P. alkekengi* mainly focused on immune cells and *Trypanosoma* infection. Previous studies utilizing concanavalin A (Con A)-activated spleen cells suggested that physalin B inhibited Con A-induced lymphoproliferation, mixed lymphocyte reaction (MLR), and IL-2 production [88]. Yu et al. [90] found that physalin H also significantly inhibited the proliferation of Con A-induced T cells and MLR in vitro, with IC_50_ values of 0.69 and 0.39 μg/mL, respectively. In vivo, physalin H dose-dependently inhibited CD4+ T cell-mediated delayed-type hypersensitivity reactions and antigen-specific T-cell response in ovalbumin-immunized mice, with IC_50_ values of 3.61 μg/mL for 48 h and 2.75 μg/mL for 96 h. The mechanisms may be related to the modulation of T-helper 1/T-helper 2 (Th1/Th2) cytokine balance, inhibition of T cell activation, and proliferation and induction of HO-1 in T cells. Moreover, at the concentration of 40 µg, polysaccharides from fruits of *P. alkekengi* showed good immunosuppressive effects in mice [91]. Physalin B decreased the number of *T. cruzi* Dm28c and *T. cruzi* transmission in the gut at doses of 1 mg/mL (oral administration), 20 ng (topical application), and 57 ng/cm^2^ (contact treatment), and suppressed epimastigote forms of *T. cruzi*, with an IC_50_ value of 5.3 ± 1.9 μM [85,87]. At a concentration of 1 μg/mL, physalin B significantly increased the mortality rate (78.1%) among *Rhodnius prolixus* larvae infected with *Trypanosoma rangeli* [86]. Physalin F prevented the rejection of allogeneic heterotopic heart transplants in vivo in a concentration-dependent manner. Moreover, it inhibited the spontaneous proliferation of peripheral blood mononuclear cells in patients with human T-cell lymphotropic virus type 1-related (HTLV1-related) myelopathy at 10 μM, suggesting its potential for treatments of pathologies in the inhibition of immune responses [88,89].

### 4.4. Antibacterial Activity

**Figure 6:**
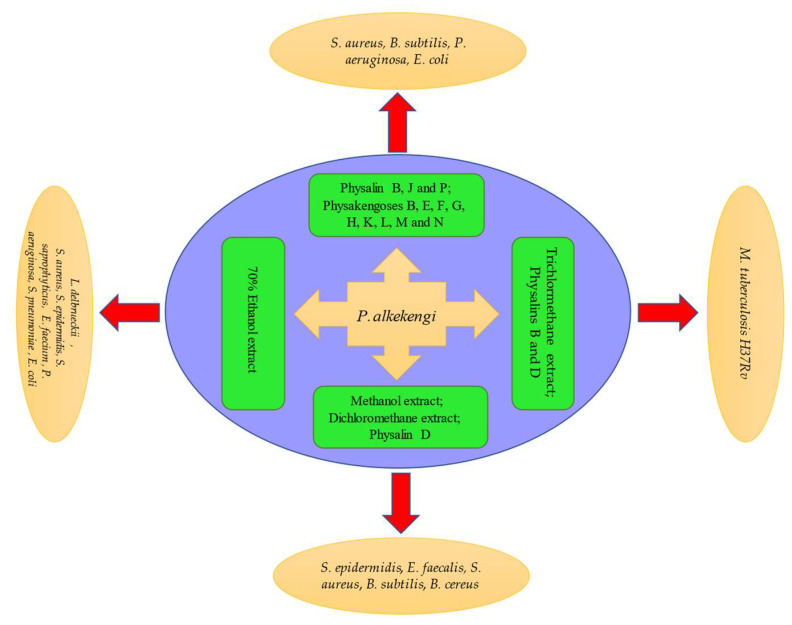
Schematic representation of antibacterial activity of *P. alkekengi* and its constituents.

In vitro, at the concentration of 100 μg/mL, physalin D isolated from *P. alkekengi* was found to be effective against *Staphylococcus epidermidis* (*S. epidermidis*), *Enterococcus faecalis* (*E. faecalis*), *Staphylococcus aureus* (*S. aureus*), and *Bacillus subtilis* (*B. subtilis*) [92]. Yang et al. [93] reported that physalins B, J, and P exhibited a good antibacterial activity against *Escherichia coli* (*E. coli*) and *B. subtilis*. Additionally, trichlormethane, ethanol, methanol, or aqueous extracts from *P. alkekengi* were also active against some Gram-positive and Gram-negative bacteria [62,94,95,96]. Janua’rio et al. [94] found that the crude trichlormethane extract (fraction A1-29-12) inhibited the *Mycobacterium tuberculosis* H37RV strain at a minimum concentration of 32 μg/mL. Li et al. [95] found that the 70% ethanol extract stimulated the growth of probiotic bacteria (*Lactobacillus delbrueckii*) and inhibited that of pathogenic bacteria (*E. coli*) in a dose-dependent manner. Moreover, a study indicated that physakengoses also have potent antibacterial activity against *S. aureus*, *B. subtilis*, and *Pseudomonas aeruginosa* (*P. aeruginosa*). The minimum inhibitory concentration (MIC) values of physakengoses B, E, F, G, and H for *S. aureus* were 9.72 ± 2.83, 9.81 ± 1.48, 5.32 ± 1.47, 6.57 ± 0.86, and 5.78 ± 0.96 μg/mL, respectively. For *B. subtilis*, these values were 8.89 ± 1.63, 5.59 ± 0.85, 3.50 ± 1.49, 8.78 ± 1.67, and 3.57 ± 1.02 µg/mL, respectively. For *P. aeruginosa*, these values were 14.91 ± 2.56, 13.12 ± 2.42, 5.79 ± 1.15, 4.51 ± 3.02, and 3.21 ± 0.95 μg/mL, respectively [96]. Zhang et al. showed that physakengoses K, L, M, N, and O had potent antibacterial activity, with MIC values ranging from 2.16 to 12.76 mg/mL [97]. However, the mechanism involved in the antibacterial activity of *P. alkekengi* has not been reported yet, warranting further research. The antibacterial activity is illustrated in Figure 6.

### 4.5. Antileishmanial Activity

Physalins exhibit potent antileishmanial activity against the cutaneous leishmaniasis [109,110]. Guimarães et al. [98] reported that physalins B and F exerted in vivo antileishmanial effects in BALB/c mice infected with *Leishmania* *amazonensis* (*L. amazonensis*); in vitro, they demonstrated an effect against intracellular amastigotes of *Leishmania*. In vitro, physalins B and F inhibited the infection of macrophages with *L. amazonensis*, with IC_50_ values of 0.21 and 0.18 μM, respectively. Physalin F markedly reduced the lesion size and number of parasites in vivo. However, physalin D did not show this activity. This effect was associated with the inhibition of NO and proinflammatory cytokines (e.g., IL-12 and TNF-α) by physalins B and F; however, physalin D lacked immunomodulatory/anti-inflammatory activity [48,88]. Meanwhile, the results suggest that anti-inflammatory and antileishmanial activities by physalins play a role in the treatment of cutaneous leishmaniasis.

### 4.6. Others

The anti-asthmatic activity of physalins has been increasingly reported over the years. In an in vitro study, following the oral administration of a water extract from *P. alkekengi*, the number of white blood cells and eosinophils in mice, as well as the expression of IL-5 and IFN-γ in lung tissue, were reduced. These findings indicated its potency as a drug for the treatment of allergic asthma in children [99]. Moreover, some studies showed that luteolin effectively inhibited inflammation in asthmatic models [111]. The relevant mechanisms may be related to the inhibition of iNOS/NO signaling. Thus, more studies are required to explain the mechanisms involved in the anti-asthmatic activity of the *P. alkekengi* extract.

Thus far, most scientific investigations on the anti-diabetic activity of *P. alkekengi* have been carried out using the fruits, aerial parts, and polysaccharides obtained from the calyxes of *P. alkekengi*. For the fruits and aerial parts, the ethyl acetate extract effectively decreased the levels of fasting blood glucose (FBG), total cholesterol (TC), triglyceride (TG), and glycated serum protein, whereas it significantly increased those of fasting insulin (FINS) [100,102]. Moreover, polysaccharides showed anti-hyperglycemic activity on alloxan-induced mice. Although research is currently at a preliminary stage, the possible mechanisms are related to the enhancement of PI3K, Akt, and glucose transporter type 4 (GLUT4) mRNA expression, as well as the inhibition of FNG and GSP expression, indicating that they are promising candidates for the development of new anti-diabetic agents [101].

The anti-ulcer and anti-*Helicobacter pylori* effects are newly discovered pharmacological effects of *P. alkekengi*. Wang et al. reported that the *P. alkekengi* extract showed anti-*Helicobacter pylori* and gastroprotective activities by reducing the intensity of gastric mucosal damage and mitigating pain sensation [63]. It was recently reported that the 70% ethanol extract of *P. alkekengi* treated LPS-induced acute lung injury by: (1) reducing the release of TNF-α and the accumulation of oxidation products; (2) decreasing the levels of NF-κB, phosphorylated-p38, ERK, JNK, p53, caspase 3 (CASP3), and COX-2; and (3) enhancing the translocation of Nrf2 from the cytoplasm to the nucleus [103]. It was also shown that the mechanism of *P. alkekengi*, which is involved in the improvement of oxidative stress damage and inflammatory response induced by acute lung injury, was related to the inhibition of NF-κB and the MAPK signaling pathway and the transduction of the apoptotic pathway, as well as the activation of the Nrf2 signaling pathway. Physalin B could be used in the treatment of dextran sulfate sodium-induced colitis in BALB/c mice by suppressing multiple inflammatory signaling pathways [50]. In addition, physalin B is effective against Alzheimer’s disease through downregulation of β-amyloid (Aβ) secretion and beta-secretase 1 (BACE1) expression by activating forkhead box O1 (FoxO1) and inhibiting STAT3 phosphorylation [104]. In the diphenyl-2-picrylhydrazyl (DPPH) and thiobarbituric acid (TBA) test, physalin D showed antioxidant activity, with an IC_50_ value ≥10 ± 2.1 μg/mL [92]. Physalins B, D, F, and G showed low anti-plasmodial activity; nevertheless, physalin D markedly caused parasitemia and a delay in mortality in mice infected with *Plasmodium berghei* [105]. Furthermore, a study demonstrated that 75% ethanol extract of calyxes and fruits of *P. alkekengi* significantly decreased the serum’s total cholesterol and TG levels in vivo. Moreover, luteolin-7-*O*-β-d-glucopyranoside isolated from *P. alkekengi* decreased the TG levels induced by oleic acid in HepG2 cells and by high glucose in primary mouse hepatocytes, thereby exhibiting hypolipidemic activity [106]. Luteolin effectively relaxed the blood vessels and preserved the rat heart, mainly through activation of the PI3K/Akt/NO signaling pathway and enhancement of the activity of endothelial NOS, as well as amelioration of the Ca^2+^ overload in rat cardiomyocytes [107,108].

## 5. Pharmacology

### 5.1. Physalins

Absorption refers to the process by which the drug enters the blood circulation from the site of administration. Following the oral administration of the extract from the calyxes and fruits of *P. alkekengi* (0.5 g/mL) in rats, liquid chromatography with MS/MS was used to investigate the pharmacokinetic profile of physalins A, D, and L (equivalent to 2, 16, and 3 mg/mL, respectively) in plasma. The results showed similar pharmacokinetic parameters for the three physalin compounds (maximum concentration: 1.3, 1.7, and 1.3 h, respectively). The biological half-life was 2.5, 3.4, and 2.8 h; the mean residence time was 3.6, 4.9, and 4.1 h; and the area under curve was 113, 103, and 266 ng∙h/mL, respectively. These data revealed that the absorption characteristics of these three physalin compounds in rats were similar. Moreover, chemical structural changes in the three compounds exerted a minimal effect on the absorption rate but a greater effect on the elimination rate [112]. This is attributed to the high degree of similarity between the chemical structures of the three physalin compounds. Another study also showed that physalins A, D, and L exhibited great similarity in the time required to reach the peak concentration (0.7, 1.2, and 0.7 h, respectively) in rat plasma. However, isophysalin B was rarely absorbed in rats due to the conversion of gastrointestinal bacteria and metabolic enzymes through a strong first-pass effect after oral administration and its low solubility in gastrointestinal fluid [113,114,115]. Pharmacokinetic studies of physalins incubated with intestinal bacterial culture showed that the concentration was significantly decreased. Furthermore, most physalins could not be detected when the reaction time was increased. These results indicated that physalins are extremely unstable in rat intestinal bacteria and have low bioavailability [115].

Distribution refers to the process by which the drug is absorbed into the blood circulation and transported to the various organs and tissues of the body. Zheng et al. [116] revealed that, after a single intragastric administration, physalin B exhibited a single-chamber model with peak concentration of 0.08 h and body clearance rate by bioavailability of 0.18 L/min/kg; the distribution decreased in the following order: C_lung_ > C_heart_ > C_kidney_ > C_brain_ > C_liver_ > C_spleen_. The concentration of physalin B in the lung was >20-fold higher than that measured in all other tissues, indicating that the lung is the main target organ of physalin B. Physalin B also showed significant antitumor activity against lung cancer cell lines (IC_50_ value: 1.2 μM) and became a therapeutic candidate for this disease [117]. Wu et al. [118] found that physalin D was distributed and rapidly eliminated in rats within 5 min, and the distribution characteristics in tissue decreased in the following order: C_kidney_ > C_liver_ > C_lung_ > C_spleen_ > C_heart_. The highest levels were recorded in the kidney, followed by the liver; however, physalin D was not detected in the brain. Therefore, kidney is the major distribution tissue for physalin D in rats, and physalin D cannot cross the blood–brain barrier. This is probably because the polarity of physalin B is lower than that of physalin D, making it easier for physalin B to cross the cell membrane than physalin D.

Metabolism is also known as biotransformation; it refers to the change in the chemical structure of the drug in the body. The main metabolic reactions of physalins in the body are phase II metabolic reactions (e.g., sulfonation, acetylation, glucuronidation, etc.). Following the oral administration of calyxes and fruits of *P. alkekengi*, an analytical method based on UHPLC-Q-TOF-MS/MS was applied to identify absorbed constituents and in vivo metabolites in biological fluids obtained from rats. The results identified 33 compounds in vivo: 12 and 21 compounds were predicted to be prototype components and metabolites of *P. alkekengi*, respectively. Lastly, sulfonation and hydroxylation were recognized as the metabolic pathways for physalin constituents [119]. Another study focused on the metabolism of physalin A in rats after oral administration. A total of 24 proposed metabolites were identified in the plasma, bile, urine, and feces. The major metabolic pathways of physalin A in the body were sulfonation, reduction, and hydroxylation. These analyses provided a framework for studying the possible metabolic pathways of other physalins and evaluating the relationship of metabolites with parent compounds in the context of the internal environment [120].

Excretion refers to the process through which the prototype of a drug or its metabolites are transported out of the body through excretory or secretory organs. A rapid and sensitive method was developed to investigate urine and feces samples collected at different exposure times after the oral administration of physalin D (25 mg/kg). The analysis showed that 12.26% of the orally administered dose of physalin D was excreted in the feces, in an unchanged form, within 72 h. The physalin D in feces was mainly excreted within 12–24 h, and the excretion ratio in feces decreased in parallel with the decreasing concentration of physalin D in the rat. Physalin D in urine was mainly excreted in the form of glucuronide and sulfate, mainly within 4–36 h, and the amount decreased with time. The excretion data of physalin D in urine and feces indicated that <14.0% of the administered dose was excreted in an unconverted form. These results revealed that physalin D was extensively and rapidly metabolized in rats after intragastric administration, leading to a short biological half-life [121]. The pharmacokinetics of physalins are shown in Table 3.

### 5.2. Flavonoids

Flavonoids are widely distributed in the calyxes and fruits of *P. alkekengi* and exhibit anti-allergic, anti-inflammatory, antioxidant, and inhibitory effects on NO as the main active ingredient [122]. However, the in vivo absorption of flavonoids has been rarely investigated. Guo et al. [112] conducted a pharmacokinetic characterization of luteolin-7-*O*-glucopyranoside and luteolin. They found that the plasma concentrations of two flavonoids could not reach the lower limit of quantitation at most timepoints. Small amounts of compounds were detected due to the relatively low levels of flavonoids (1 and 0.4 mg/g, respectively) in extracts from the calyxes and fruits of *P. alkekengi*. Moreover, evidence suggested that flavonoids were usually consumed in the small intestine as a proportion of aglycone [123]. Luteolin is produced by the metabolism of luteoloside. Subsequently, it may be transported to the liver through the portal vein, where it may form a potential phase I substrate through further hydroxylation in the liver. Consequently, it produces more polar compounds through further phase I and II metabolism [124,125], such as the glucuronidation of luteolin and the hydroxylation and sulfation of other types of flavonoids [119]. Therefore, the content of prototype constituents in the body would be significantly decreased or undetectable.

## 6. Conclusions and Future Perspectives

Thus far, >170 compounds have been isolated and identified from *P. alkekengi*; the most common are physalins, flavonoids, sucrose esters, and other trace elements [5]. Among these ingredients, 18 new compounds were isolated from the *P. alkekengi*, including nine steroids (7α-hydroxy-5-deoxy-4-dehydrophysalin IX, 5-deoxy-4-dehydrophysalin IX, 7β-ethoxyl-isophysalin C, etc.) [77,97,126,127]. Numerous pharmacological studies have revealed various biological properties of *P. alkekengi* (i.e., anti-inflammatory, anti-cancer, immunosuppressive, anti-leishmanial, anti-asthmatic, anti-diabetic, antioxidative, anti-malarial, anti-vasodilatory, anti-colitic, anti-ulcer, acting as febricide, expectorant, or diuretic, etc.). Physalins and flavonoids are closely related to the pharmacological activity of *P. alkekengi*. Accordingly, further study is urgently needed to gain a better understanding of *P. alkekengi* and its clinical use.

Firstly, this review summarizes the structural analysis of natural products of physalins and flavonoids. Physalins are synthesized in *P. alkekengi* via MEV and MEP pathways, and flavonoids are synthesized via phenylpropanoid pathway. However, apart from some studies on the physalins’ skeleton of natural products, there is almost no research conducted on the synthesis of specific physalins and their derivatives. The importance of the right-side (DFGH-ring) structure of physalins for biological activity is established. For example, synthesis of DFGH-ring derivatives of physalins with a hydrophobic substituent is important for the inhibitory activity [128]. Therefore, there is a need for further exploration of the synthesis of physalins and development of more clinically valuable compounds.

Secondly, a holistic quality control method that is correlated with the pharmacological effects of *P. alkekengi* is warranted. Current quality control methods are mainly focused on HPLC, and it is difficult to distinguish genuine products from counterfeit goods. The purpose of quality control in TCM is to monitor effective substances and their variations in the production process. By summarizing the currently available literature, we found that the contents of bioactive compounds differ significantly in samples obtained from different sources and at different collection times. Therefore, safe, high-quality, and high efficiency planting techniques for this plant should be further investigated to guide its production for TCM.

Thirdly, previous pharmacological investigations on *P. alkekengi* have yielded considerable evidence regarding its anti-inflammatory and anti-cancer properties and have elucidated the mechanisms of their action in vitro and in vivo. However, few studies concentrated on its immunosuppressive, anti-leishmanial, anti-asthmatic, anti-diabetic, antioxidative, anti-malarial, anti-vasodilatory, and anti-colitic effects, which warrant further exploration. Additionally, several studies have highlighted the potential of *P. alkekengi* as a novel therapeutic agent for the treatment of ulcers, *Helicobacter pylori*, LPS-induced induced acute lung injury, and Alzheimer’s disease. Nevertheless, the mechanism underlying these treatment effects should be fully elucidated using current techniques.

Lastly, the absorption, distribution, metabolism, and excretion of physalins in the body are explained. These compounds are characterized by fast absorption, wide distribution, and rapid excretion. These findings indicated that physalins are extremely unstable and have low bioavailability in the intestine. Hence, they must overcome certain factors that control the sustained and stable release of drugs in the blood and improve oral bioavailability, thereby exerting good pharmacological effects. Unfortunately, however, it should be noted that few studies have investigated the pharmacokinetics of extracts and active compounds, particularly flavonoids. Consequently, further clinical application of *P. alkekengi* may be limited until further pharmacokinetics studies in the laboratory and clinic are performed.

In summary, *P. alkekengi* is an excellent, abundant, inexpensive, and edible drug. The synthesis of the main active components of *P. alkekengi* must be further analyzed using additional biological and chemical techniques to further expand their potential applications. In addition, the quantitative analysis of the chemical constituents of *P. alkekengi* should be employed for the purpose of standardization and quality control of extracts. Lastly, additional in vivo animal research and clinical trials are needed to determine whether various applications of *P. alkekengi* are effective and safe in a larger population.

## Figures and Tables

**Table 1:** Quantitative analysis for the quality control of *P. alkekengi*.

Analytes	Method	Part Used	Results	Reference
Physalins A, O, L, and B	HPLC	Fruits and calyxes	In 10 habitats: 1.04–3.12, 0.99–2.66, 0.59–0.91, and 0.54–1.31 mg/g, respectively.	[32]
Physalins B, D, G, and H, 4,7-didehydroneophysalin B	UPLC-MS/MS	Fruits and calyxes Calyxes	In 14 habitats: 30.75–749.13, 59.63–1046.63, 15.25–527.15, 1.00–254.05, 15.75–70.88 μg/g, respectively 467.84, 560.34, 352.06,156.69, 43.22 μg/g, respectively	[39]
Physalin D	RP-HPLC–UV	Calyxes Fruits	In mature and immature: 0.2028 ± 0.0160%, 0.7880 ± 0.0612%, respectively In mature and immature: 0.0259 ± 0.0021%, 0.0992 ± 0.0083%, respectively	[35]
4,7-didehydroneophysalin B	HPLC	Fruits	0.02%	[40]
4,7-didehydroneophysalin B	HPLC	Fruits and calyxes	50% and 70% ethanol extract: 2.18%, 0.42%, respectively	[41]
Physalins A, P and O, Luteoloside, luteolin	HPLC	Fruits	In 6 habitats: 0.048–0.24, 0.04–0.2, 0.36–1.8, 0. 052–0.26, 0.04–0.2 μg/mL, respectively	[33]
Luteoloside	TLC	Fruits and calyxes	In 11 habitats: 0.11–2.27 mg/g	[42]
Luteolin	HPLC	Physalis permviana liquid	0.75 μg/mL	[45]
Polysaccharides Reducing sugar	UV	Calyxes Fruits	In 52 habitats: 0.34–9.67, 1.32–146.53 mg/g, respectively In 50 habitats: 2.47–11.82, 181.97–321.57 mg/g, respectively	[43]
Luteoloside Luteolin	HPLC	Jinhuang yanyan tablets	0.14%–0.15%, 0.0066%–0.0070%, respectively	[46]
Lutein β-carotene	HPLC-DAD-APCI-MS	Fruits	19.8–21.6 mg/100 g of total lutein and β-carotene contents	[44]
Citric acid Malic acid Tartaric acid Ascorbic acid	HPLC-UV	Fruits	903–920 mg/100 g 396–554 mg/100 g 261–325 mg/100 g 26–32 mg/100 g	[5]
(hydroxy)cinnamoyl hexosides Sinapoyl Feruloyl hexosides	HPLC-DAD-ESI-MS	Fruits	70.8–81.6 mg/kg 57.8–68.0 mg/kg 10.6–13.6 mg/kg	[5]
Aromatic amino acids and amino derivatives	HPLC-DAD-ESI-MS	Fruits	50.9–63.5 mg/kg	[5]

Abbreviations: APCI, atmospheric pressure chemical ionization; DAD, diode array detection; ESI, electrospray ionization interface; HPLC, high-performance liquid chromatography; MS, mass spectrometry; MS/MS, tandem mass spectrometry; RP, reverse phase; TLC, thin-layer chromatography; UPLC, ultra-performance liquid chromatography; UV, ultraviolet.

**Table 2:** Pharmacological effects of *P. alkekengi*.

Pharmacological Activity	Animal/Cell Models	Constituent/Extract	Detail	Dosage	Reference
Anti-inflammatory activity	LPS-induced 264.7 cells	Physalins A, O, L, G Isophysalin A	Induced NO production	20 μM	[47]
	IFN-γ-stimulated macrophages LPS-stimulated macrophages	Physalins B, F, G	Reduced NO production; inhibited TNF-α, IL-6, IL-12	2 μg/mL	[48]
	C57BL/6 mice	Physalins B, F	Suppressed the increase in TNF-α; increased vascular permeability; prevented neutrophil influx	20 mg/kg	[49]
	LPS-induced 264.7 cells	Physalin B	Decreased the levels of TNF-α, IL-6, IL-1β	0.25, 0.5, 1.0 μM	[50]
	LPS/IFN-γ-induced macrophages IL-4/IL-13-induced macrophages LPS-induced C57BL/6 mice	Physalin D	In vitro: activated signal transducer and activator of STAT6 pathway; suppressed STAT1 activation; blocked STAT1 nuclear translocation In vivo: reduced inducible iNOS cell number; increased CD206+ cell number	5 μM	[51]
	LPS-stimulated RAW 264.7 cells	Physalin E	Inhibited the generation of TNF-α, IL-6, NF-κB p65; reduced the degradation of I-kappa B protein	12.5, 25, 50 μM	[52]
	TPA-induced acute ear edema in mice Oxazolone-induced chronic dermatitis in mice	Physalin E	Reduced ear edema response and myeloperoxidase activity; suppressed increase in ear thickness and levels of TNF-α and IFN-γ	0.125, 0.25, 0.5 mg/ear	[53]
	DBA/1 mice	Physalin F	Decreased paw edema and joint inflammation	60 mg/kg	[54]
	LPS-induced macrophages	Physalin X Aromaphysalin B	Inhibited NO production	IC_50_ = 68.50, 29.69 μM, respectively	[55]
	LPS-induced macrophages	Physalins B, F, H, V, D1, VII, I Isophysalin B	Inhibited NO production	IC_50_ = 0.32–4.03, 12.83–34.19 μM, respectively.	[56]
	LPS-induced macrophages	Physalins A, B, F Ombuine Luteolin	Inhibited NO production	IC_50_ = 2.57 ± 1.18, 0.84 ± 0.64, 0.33 ± 0.17, 2.23 ± 0.19, 7.39 ± 2.18 µM, respectively.	[57]
	LPS/IFN-γ-stimulated macrophages ICR mice	Luteolin	In vitro: suppressed the production of IL-6, IL-12, and TNF-α In vivo: inhibited paw edema	20 μM 20 mg/kg	[58]
	KF-8 cells	Apigenin Lutelin	Inhibited NF-κB activation and the expression of CCL2/MCP-1 and CXCL1/KC	20 μM	[59]
	LPS-induced macrophages	Kaempferol Quercetin	Inhibited STAT-1 and NF-κB activation, iNOS protein and mRNA expression, and NO production	100 μM	[60,61]
	LPS-stimulated THP-1 cells ICR mice	70% ethanol extract	In vitro: reduced the production of NO, PGE2, TNF-α, IL-1, iNOS, and COX-2 In vivo: reduced ear edema; induced granulomatous tissue formation	500 μg/mL	[62]
	Wistar rats	Methanol extract	Reduced the paw volume	500 mg/kg	[63]
	LPS-induced macrophages	Physanosides B	Inhibited NO production	IC_50_ = 9.93 μM	[64]
	LPS-induced macrophages	(6S,9R)-roseoside	Inhibited NO production	IC_50_ = 7.31 μM	[65]
Anti-tumor activity	HepG2 cells	Physalin A	Activated the Nrf2–ARE pathway and its target genes	20 μM	[65]
	Non-small cell lung cancer BALB /c mice	Physalin A	In vitro: suppressed both constitutive and induced STAT3 activity In vivo: suppressed tumor xenograft growth	5,10, 15 μM 40, 80 mg/kg	[66]
	Human melanoma A375-S2 cells	Physalin A	Activated transmembrane death receptor; Induced poptosis via apoptotic (intrinsic and extrinsic) pathway; up-regulated p53-NOXA-mediated ROS generation	15 μM	[67]
	Human HT1080 fibrosarcoma cells	Physalin A	Upregulated CASP3, CASP8 expression	IC_50_ = 10.7 ± 0.91 μM	[68]
	Human melanoma A375-S2 cells	Physalin A	Repressed the production of RNS and ROS; triggered the expression of iNOS and NO	15 μM	[69]
	Non-small cell lung cancer	Physalin A	Induced G2/M cell cycle arrest; increased the amount of intracellular ROS	IC_50_ = 28.4 μM	[70]
	Prostate cancer cells (CWR22Rv1, C42B)	Physalins A, B	Inhibited the growth of two cells; activated the JNK and ERK pathway	IC_50_ = 14.2, 9.6 μM, respectively	[71]
	Non-small cell lung cancer	Physalin B	Exhibited anti-proliferative and apoptotic activity; downregulated the CDK1/CCNB1 complex; upregulated p21	5, 10, 20 μmol/L	[72]
	Human melanoma A375 cells	Physalin B	Activated the expression of the NOXA, BCL2 associated X (Bax), and CASP3	3 μg/mL	[73]
	Human HCT116 colon cancer cells	Physalin B	Activated the ERK, JNK, and p38 MAPK pathways; increased ROS generation	IC_50_ = 1.35 μmol/L	[74]
	Human DLD-1 colon cancer cells	Physalin B	Inhibited TNFα-induced NF-κB activation; induced the proapoptotic protein NOXA generation	5 μM	[75]
	Breast cancer cells (MCF-7, MDA-MB-231, T-47D)	Physalin B	Induced cell cycle arrest at G2/M phase; promoted the cleavage of PARP, CASP3, CASP7, and CASP9; inactivated Akt and P13K phosphorylation	2.5, 5, 10 μM	[76]
	TNF-α-stimulated HeLa cells	Physalins B, C, F	Inhibited the phosphorylation and degradation of IκBα and NF-κB activation	IC_50_ = 6.07, 6.54, 2.53 μM, respectively	[9]
	Tumor cells (A549, K562)	(17S,20R,22R)-5β,6β-epoxy-18,20-dihydroxy-1-ox- owitha-2,24-dienolide withaphysalin B	Suppressed the PI3K/Akt/mTOR signaling pathway	IC_50_ = 1.9–4.3 μM	[77]
	Tumor cells (B-16, HCT-8, PC3, MDA-MB-435, MDA-MB-231, MCF-7, K562, CEM, HL-60) Swiss mice	Physalins B, D	In vitro: displayed activity against several cancer cell lines In vivo: inhibited the proliferation of cells; reduced Ki67 staining	0.58–15.18, 0.28–2.43 μg/mL, respectively 10, 25 mg/kg	[78]
	Human cancer cells (C4-2B, 22Rv1, 786-O, A-498, ACHN, A375-S2)	Physalins B, F	Showed anti-proliferative activities	IC_50_ = 0.24–3.17 μM	[56]
	Human T cell leukemia Jurkat cells	Physalins B, F	Inhibited PMA-induced NF-κB and TNF-α-induced NF-κB activation	8, 16 µM, respectively	[79]
	HEK293T cells BALB/c-nu/nu mice	Physalin F	In vitro: decreased TOPFlash reporter activity; promoted the proteasomal degradation of β-catenin In vivo: downregulated β-catenin	4 μM 10, 20 mg/kg	[80]
	T-47D cells	Physalin F	Activated the CASP3 and c-myc pathways	IC_50_ = 3.60 μg/mL	[81]
	Human renal, carcinoma cells (A498, ACHN, UO-31)	Physalin F	Induced cell apoptosis through the ROS-mediated mitochondrial pathway; suppressed NF-κB activation	1, 3, 10 μg/mL	[82]
	PC-3 cancer cell lines	7β-ethoxyl-isophysalin C	Showed apparent moderate activities	IC_50_ = 8.26 µM	[83]
	Human osteosarcoma cells	Physakengose G	Inhibited the epidermal growth factor receptor/mTOR (EGFR/mTOR) pathway; blocked autophagic flux through lysosome dysfunction	5, 10, 20 μM	[84]
Immunosuppressive activity	*Trypanosoma cruzi (T. cruzi)*-infected insects	Physalin B	Decreased number of *T. cruzi* Dm28c and *T. cruzi* transmission; inhibited the development of parasites	1 mg/mL 20 ng 57 ng/cm^2^	[85]
	H14 *Trypanosoma rangeli*-infected *Rhodnius prolixus* larvae	Physalin B	Reduced the production of hemocyte microaggregation and NO	0.1, 1 μg/mL	[86]
	*T. cruzi* trypomastigotes BALB/c mice macrophages	Physalin B Physalin F	Displayed strongest effects against epimastigote forms of *T. cruzi*	IC_50_ = 5.3 ± 1.9, 5.8 ± 1.5 μM, respectively IC_50_ = 0.68 ± 0.01, 0.84 ± 0.04 μM, respectively	[87]
	Con A-induced spleen cells CBA mice	Physalins B, F, G	In vitro: inhibited MLR and IL-2 production In vivo: prevented the rejection of allogeneic heterotopic heart transplant	2 μg/mL 1 mg/mouse/day	[88]
	Human T-cell lymphotropic virus type 1 (HTLV-1)-infected subjects	Physalin F	Inhibited spontaneous proliferation; reduced the levels of IL-2, IL-6, IL-10, TNF-α, and IFN-γ	10 μM	[89]
	T cells BALB/c mice	Physalin H	In vitro: suppressed proliferation and MLR In vivo: inhibited delayed-type hypersensitivity reactions and T-cell response	IC_50_ = 0.69, 0.39 μg/mL, respectively IC_50_ = 2.75 or 3.61 μg/mL	[90]
	ICR mice	Polysaccharides	Enhanced specific antibody titers immunoglobulin G (IgG), IgG1, and IgG2b, as well as the concentration of IL-2 and IL-4	40 µg/mice	[91]
Anti-microbial activity	Gram-positive bacteria: *Staphylococcus epidermidis* (*S. epidermidis*), *Enterococcus faecalis* (*E. faecalis*)*, Staphylococcus aureus* (*S. aureus*), *Bacillus subtilis* (*B. subtilis*), *Bacillus cereus* (*B. cereus*)	Methanol extract Dichloromethane extract Physalin D	Displayed moderate antibacterial activity	MIC = 32–128 µg/mL	[92]
	*Escherichia coli* (*E. coli*)*, B. subtilis*	Physalins B, J, P	Showed high antibacterial activity	MIC = 12.5–23.7, 23.23–24.34, 22.8–27.98 µg/mL, respectively	[93]
	*Mycobacterium tuberculosis* H37Rv	Trichlormethane extract Physalins B, D	Showed antibacterial activity	MIC = 32, >128, 32 µg/mL, respectively	[94]
	*Lactobacillus delbrueckii* (*L. delbrueckii*), *E. coli*	70% ethanol extract	Promoted the growth of *L. delbrueckii*; inhibited the growth of *E. coli*	0.78–1.56 mg/mL	[95]
	Gram-positive bacteria: *S. aureus, S. epidermidis, Staphylococcus saprophyticus* (*S. saprophyticus*)*, Enterococcus faecium* (*E. faecium*) Gram-negative bacteria: *Pseudomonas aeruginosa* (*P. aeruginosa*)*, Streptococcus pneumoniae* (*S. pneumoniae*), *E. coli*	70% ethanol extract	Showed antibacterial activity	MIC = 0.825–1.65 mg/mL	[62]
	*S. aureus, B. subtilis, P. aeruginosa, E. coli*	Physakengoses B, E, F, G, H, K, L, M, N, O	Showed potent inhibitory effects	MIC = 2.16–14.9 μg/mL	[96,97]
Anti-leishmanial	*Leishmania*-infected macrophages *Leishmania amazonensis* -infected BALB/c mice	Physalins B, F	In vitro: reduced the percentage of macrophages In vivo: reduced the lesion size, the parasite load, and histopathological alterations	IC_50_ = 0.21 and 0.18 μM, respectively	[98]
Others	Kunming mice	Water extract	Decreased the expression of white blood cells and eosinophils, IL-5, IFN-γ, Th1, and Th2	0.25, 5, 1 g/mL	[99]
	3T3-L1 pre-adipocyte cells HepG2 cells Male Sprague–Dawley (SD) rats	Ethyl acetate extract	In vitro: relieved oxidative stress; inhibited α-glucosidase activity. In vivo: decreased FBG, TC, and TG	300 mg/kg	[100]
	Alloxan-induced mice	Polysaccharides	Decreased FBG and GSP; increased FINS; upregulated the PI3K, Akt, and GLUT4 mRNA	200, 400, 800 mg/kg	[101]
	High-fat diet-fed and streptozotocin-induced diabetic SD rats	Ethyl acetate extract	Reduced the FBG, TC, TG, and GSP; increased FINS	300, 600 mg/kg	[102]
	Wistar rats Albino mice	Aqueous methanolic extract	Reduced the intensity of gastric mucosal damage; inhibited pain sensation	500 μg/mL 500 mg/kg	[63]
	LPS-induced acute lung injury in BALB/c mice	70% ethanol extract	Reduced the release of TNF-α and the accumulation of oxidation products; decreased the levels of NF-κB, phosphorylated-p38, ERK, JNK, p53, CASP3, and COX-2	500 mg/kg	[103]
	4% dextran sulfate sodium--induced colitis in BALB/c mice	Physalin B	Reduced MPO activity; suppressed the activation of NF-κB, STAT3, arrestin beta 1 (ARRB1), and NLR family pyrin domain containing 3 (NLRP3)	10, 20 mg/kg	[50]
	N2a/APPsw cells	Physalin B	Downregulated β-amyloid (Aβ) secretion and the expression of beta-secretase 1 (BACE1)	3 μmol/L	[104]
	DPPH TBA	Physalin D	Exhibited antioxidant activity	IC_50_ ≥ 10 ± 2.1 µg/mL	[92]
	*Plasmodium berghei*-infected mice	Physalins B, D, F, G	Caused parasitemia reduction and delay	50, 100 mg/kg	[105]
	High glucose-induced primary mouse hepatocytes Oleic acid-induced HepG2 cells Kunming mice	75% ethanol extract Luteolin-7-*O*-β-d-glucopyranoside	In vitro: decreased the levels of TG in HepG2 cells In vivo: decreased the levels of TC and TG	50, 100 μg/mL, respectively 1 or 2 g/kg, 0.54 g/kg, respectively	[106]
	SD mice	Luteolin	Increased NO; activated PI3K/Akt/NO signaling pathway; enhanced the activity of endothelial NOS	7.5 µg/mL	[107]
	SD rats	Luteolin	Conferred a cardioprotective effect; ameliorated Ca^2+^ overload	7.5, 15, 30 μmol/L	[108]

**Table 3:** Summary of the pharmacokinetic parameters of physalins in rat plasma after single oral administration of *P. alkekengi*.

Methods	Compounds	Dose/ mg/kg	t_1/2_/h	C_max_/ ng/mL	T_max_/h	CL/L/min/kg	MRT_0-t_/ h	MRT_0-∞_/h	AUC_0-t_/ ng·h/mL	AUC_0-∞_/ ng·h/mL	Reference
LC-MS/MS	Physalin A Physalin D Physalin L	2 16 3	2.52 ± 0.40 3.36 ± 0.26 2.82 ± 0.25	5.30 ± 1.76 11.5 ± 3.57 56.4 ± 15.4	1.29 ± 2.31 1.67 ± 1.46 1.28 ± 1.33	- - -	3.63 ± 0.57 4.85 ± 0.37 4.07 ± 0.37	- -	21.0 ± 3.14 70.5 ± 10.10 200 ± 31.30	113 ± 103 103 ± 30.2 266 ± 53.0	[112]
UPLC–MS/MS	Physalin D Physalin G 4,7-Didehydroneophysalin B	35.6 13.9 32.6	3.67 ± 1.04 8.04 ± 3.42 6.15 ± 1.20	47.6 ± 4.10 20.9 ±4.40 23.6 ± 4.90	1.17 ± 0.00 1.17 ± 0.00 1.17 ± 0.00	4.4 ± 0.60 3.2 ± 0.70 8.7 ± 1.90	3.42 ± 0.33 4.69 ± 1.41 4.89 ± 0.43	- -	60.82 ± 14.32 61.24 ± 11.53 60.82 ± 12.85	136.94 ± 17.18 74.56 ± 17.46 64.82 ± 14.80	[113]
LC-MS/MS	Physalin L	18.52	2.89 ± 1.14	77.48 ± 28.30	0.69 ± 0.26	50.26 ± 11.50	3.13 ± 0.63	4.33 ± 1.50	280.78 ± 86.48	313.10 ± 101.24	[114]
HPLC-MS/MS	Physalin B	5	5.35 ± 0.49	395.0 ± 35.4	0.08 ± 0.0	0.18 ± 0.03	-	-	382.25 ± 24.87	449.92 ± 27.46	[116]
HPLC-MS/MS	Physalin D	2	0.09 ± 0.07	941.3 ± 272.1	0.08 ± 0.0	0.12 ± 0.01	0.30 ± 0.12	-	28.30 ± 29.02	283.89± 28.37	[118]
SPE-LC-MS/MS	Physalin A Physalin D Physalin G 4,7-Didehydroneophysalin B	29 38.8 18.3 31.6	1.83 ± 0.61 3.11 ± 1.37 2.24 ± 1.47 2.32 ± 1.01	12.73 ± 2.08 64.58 ± 21.30 89.93 ± 26.05 19.63 ± 7.21	0.67 ± 0.15 1.29 ± 0.78 0.67 ± 0.00 1.13 ± 0.32	-	-	-	65.21 ± 10.52 615.39 ± 97.86 159.12 ± 34.76 105.5 ± 28.21	96.31 ± 30.50 885.18 ± 230.68 205.07 ± 49.8 173.58 ± 17.90	[115]

Abbreviation: AUC, area under curve; CL, clearance rate; C_max_, maximum concentration; MRT, mean residence time; t_1/2_, biological half-life; T_max_, peak concentration.

## Data Availability

All reported or analyzed data in this review are extracted from published articles.
